# Diversity of Parvovirus 4–like Viruses in Humans, Chimpanzees, and Monkeys in Hunter–Prey Relationships

**DOI:** 10.3201/eid1805.111849

**Published:** 2012-05

**Authors:** Cornelia Adlhoch, Marco Kaiser, Anna Loewa, Markus Ulrich, Christian Forbrig, Edgard V. Adjogoua, Chantal Akoua-Koffi, Emmanuel Couacy-Hymann, Siv Aina J. Leendertz, Wolfram Rietschel, Christophe Boesch, Heinz Ellerbrok, Bradley S. Schneider, Fabian H. Leendertz

**Affiliations:** Robert Koch Institute, Berlin, Germany (C. Adlhoch, A. Loewa, M. Ulrich, C. Forbrig, H. Ellerbrok, F.H. Leendertz);; GenExpress GmbH, Berlin (M. Kaiser);; Institut Pasteur Côte d’Ivoire, Abidjan, Côte d’Ivoire (E.V. Adjogoua, C. Akoua-Koffi, E. Couacy-Hymann);; Norwegian School of Veterinary Science, Oslo, Norway (S.A.J. Leendertz);; Botanical Zoological Garden, Stuttgart, Germany (W. Rietschel);; Max-Planck-Institute for Evolutionary Anthropology, Leipzig, Germany (C. Boesch);; Global Viral Forecasting, San Francisco, California, USA (B.S. Schneider)

**Keywords:** chimpanzees, Côte d’Ivoire, colobus monkeys, partetetravirus, parvovirus 4, PARV4, PARV4-like, viruses, zoonoses, humans

## Abstract

During 2010–2011, we investigated interspecies transmission of partetraviruses between predators (humans and chimpanzees) and their prey (colobus monkeys) in Côte d’Ivoire. Despite widespread infection in all species investigated, no interspecies transmission could be detected by PCR and genome analysis. All sequences identified formed species- or subspecies *(*chimpanzee)-specific clusters, which supports a co-evolution hypothesis.

Since 2005, new parvoviruses have been discovered in the following groups: humans (parvovirus 4 [PARV4]), bats (*Eidolon helvum* parvovirus 1), and other mammals (cows, pigs, wild boars, and sheep; Hong Kong virus) (*1**–**5*). Phylogenetic analysis suggests that these parvoviruses form a separate novel genus, with the proposed name of Partetravirus, within the subfamily *Parvovirinae*. Globally, 3 genotypes of PARV4 have been found to infect humans (*6**,**7*). Recently, PARV4-like viruses have also been described in chimpanzees and gorillas (*8*). Researchers have suggested that partetraviruses have co-diverged with their hosts during mammalian evolution. Strains described so far have shown restricted sequence diversity within their host-specific clusters. However, the highly restricted sequence diversity of circulating variants of PARV4 also suggests that the virus has emerged and spread in the human population relatively recently. To clarify whether interspecies transmission is possible for primate PARV4-like viruses, as has been shown for other parvoviruses (*9*), we investigated samples in a setting where transmission of certain simian viruses between these species has been documented (*10**,**11*). We analyzed samples from wild chimpanzees (*Pan troglodytes verus*) in the Taï National Park, Côte d’Ivoire; their prey, red colobus monkeys (*Piliocolobus badius*) and black-and-white colobus monkeys (*Colobus polykomos*); and humans who hunt colobus monkeys in the same region.

## The Study

Chimpanzee and monkey samples were obtained as described from 2002–2007 (*10**,**11*). Human volunteers, recruited during a broad study of primate-borne zoonoses (samples collected between June 2006 and January 2007), ranged in age from 7 to 95 years, and all lived adjacent to the primate habitat. All human participants acknowledged eating bushmeat; most (74%) also reported butchering animals, and a small group (8%) admitted hunting bushmeat. Written informed consent forms were signed by all participants. Ethical approval was obtained from the Institut Pasteur Côte d’Ivoire and the Ministère de la Santé of Côte d’Ivoire. Cross-contamination of samples was avoided by using disposal materials and maintaining a strict safety regime for sampling humans and animals. Samples from different species were handled separately throughout the process, from sampling to analysis.

DNA from 17 chimpanzees (lung, spleen, or liver), 30 red colobus monkeys (buffy coat, blood, bone marrow, intestine, spleen, liver, lung, or muscle), and 15 black-and-white colobus monkeys (buffy coat, liver, intestine, or muscle) and 700 humans (blood), was prepared by using commercial DNA extraction kits (QIAGEN, Hilden, Germany). Partetravirus generic quantitative real-time PCR (qPCR) was used to screen the samples as described (*1*). Viral DNA from positive samples was amplified by using primers for conserved regions spanning human variants and other partetraviruses described in pigs and cows (*2*).

PARV4-like viruses were detected by qPCR in 7 (41%) of 17 chimpanzees tested. Seven (23%) of 30 red colobus and 2 (13%) of 15 black-and-white colobus monkeys were positive for PARV4-like viruses. The availability of only 1 sample per individual animal limited the analysis of viral tissue distribution (Table).

**Table:** Results of testing analyzed samples for PARV4 and PARV4-like viruses, Côte d’Ivoire*

Sample origin	Sample material, no. PCR positive/no. tested (%)							
	Blood	Bone marrow	Intestin*e*	Spleen	Muscle	Liver	Lung	Total
Red colobus monkey (*Piliocolobus badius*)	1/14 (7)†	2/6 (33)	0/1	0/3	2/2 (100)	1/2 (50)	1/2 (50)	7/30 (23)
Black and white colobus monkey (*Colobus polykomos*)	2/10 (20)‡	–	0/1	–	0/3	0/1	–	2/15 (13)
Wild chimpanzee (*Pan troglodytes verus*)	0/1‡	–	–	5/13 (38)	–	–	2/3 (67)	7/17 (41)
Human§	12/700 (2)	–	–	–	–	–	–	12/700 (2)

*Each sample represents 1 animal or person; PARV4, parvovirus 4; –, no sample. †Blood or buffy coat. ‡Buffy coat. §Dried blood spot on filter paper.

In a recent study of 91 Old World monkeys tested, none exhibited PARV4-like virus seroreactivity, whereas 63% of chimpanzees (*P.t. troglodytes*) and 18% of gorillas (*Gorilla gorilla*) were reactive (*8*). The Old World monkeys belonged to the family *Cercopithecinae*, whereas the PARV4-positive *Colobus* and *Piliocolobus* species described herein belong to the *Colobinae* branch of family *Ceropithecidae*. The serologic assay performed by Sharp et al. is based on a human PARV4 ELISA; therefore, as suggested by the study’s authors, PARV4-like viruses that infect members of the *Ceropithecinae* family might be too divergent to cross-react. The percentage (43%) of *P.t. verus* chimpanzees that tested positive for PARV4-like DNA in our study is comparable to the proportion of seropositive *P.t. troglodytes* chimpanzees in Cameroon (63%), although only 1 animal was positive by PCR in Cameroon. The low rate of PCR-positive results from serum samples in the study by Sharp et al. (*8*), compared with the rate in the current study, is not unexpected: PARV4 can be found in tissues of nonviremic animals and humans because of its persistence in tissue after resolution of acute infections.

In 12 (1.7%) of the 700 human blood samples, PARV4 genotype 3 was amplified. The median age of infected persons was 11 years (mean 20.8 years, range 7–75 years). As discussed previously (*12*), the broad age range suggests that PARV4 genotype 3 in Africa has alternative routes of infection from genotypes 1 and 2 that are found in Europe.

Near full-length nucleotide sequences from viral genomes were generated from samples from 4 persons (GenBank accession nos. JN798193–196), 1 chimpanzee (JN798203), and 1 black-and-white colobus monkey (JN798211). Partial sequences were derived from 7 persons (JN798192, JN798197–201), 3 chimpanzees (JN798204–206), and 4 red colobus monkeys (JN798207–210).

On the basis of phylogenetic analyses of the 20 sequences obtained, we could not detect any interspecies transmission; all sequences formed host species–specific clusters (Figure). Sequence diversity of genotype 3 viruses from humans in the defined region in our study was 0.9% over 1,423 nt positions, which is in accordance with what has been published (*12*). One additional near full-length genotype 3 sequence from Africa was included in the phylogenetic analysis; it clustered with human sequences found in this study. Compared with sequences from human PARV4 (AY622943) isolates, sequences from isolates from chimpanzees differed by 19% over 4,771 nt and formed a distinct cluster, together with sequences derived from chimpanzees of other subspecies. PARV4-like virus from *P.t. verus* (JN798203) from this study and *P.t. troglodytes* (HQ113143) from Cameroon (*8*) differed by 10%, suggesting subspecies-specific PARV4-like viruses within chimpanzees of 2 different subspecies from distinct habitats. Black-and-white colobus monkey viruses exhibited a closer relationship to viruses from humans than did PARV4-like viruses from red colobus monkeys. This finding corresponds to a previous phylogenetic analysis in which the genomic relationship of different species showed that colobus monkeys separated earlier from piliocolobus monkeys during evolution (*13*). The distance between PARV4-like viruses from piliocolobus monkeys to those of colobus monkeys supports these data.

**Figure:**
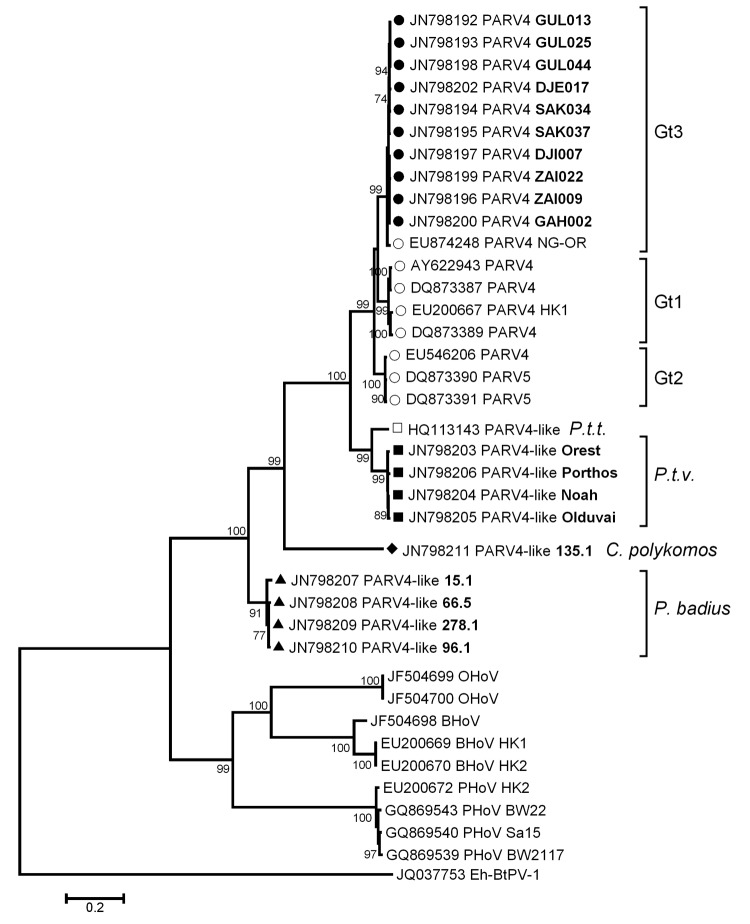
Phylogenetic tree of near full-length and partial sequences (open reading frame 2) of parvovirus 4 (PARV4), PARV4-like viruses, and Hong Kong virus (HoV) created by using MEGA5.05 (www.megasoftware.net) with the maximum likelihood-method (GTR+G+I) and bootstrap analysis of 1,000 resamplings. Sequence origin was indicated as follows: circle, from humans; square, from chimpanzees; triangle, from red colobus monkeys; and tetragon, from black-and-white colobus monkeys. New sequences from Côte d’Ivoire are shown with filled symbols. *P.t.t.*, *Pan troglodytes troglodytes*; *P.t.v.*, *Pan troglodytes verus*; *P. badius*, *Piliocolobus badius*; *C. polykomos*, *Colobus polykomos*; BHoV, bovine HoV; PHoV, porcine HoV; OHoV, ovine HoV; Eh-BtPV-1, *Eidolon helvum* parvovirus 1; Gt, genotype of human PARV4. Scale bar indicates nucleotide substitutions per site.

To evaluate whether underlying infections were present in the hunters (chimpanzees and humans) originating from the prey, a red colobus PARV4-like virus–specific qPCR was designed. Samples from humans, chimpanzees, and black-and-white colobus monkeys, which previously tested positive in the generic partetravirus qPCR, were retested.

Although chimpanzees consumed immense quantities of red colobus meat and organs (≈45 kg/year for adult males [*14*]), we could not detect red colobus PARV4-like virus in any of the 17 chimpanzees analyzed. Similarly, no red colobus PARV4-like virus DNA was discovered in humans, although in this particular region the red colobus monkey is the most hunted and eaten primate (*15*). These results support the hypothesis that PARV4-like viruses are species specific, notwithstanding constant high exposure to infectious materials.

## Conclusions

We demonstrated that partetravirus infection is widespread in monkeys, chimpanzees, and humans in West Africa. However, the PARV4-like viruses seem to remain species specific, despite continuous opportunities for interspecies transmission. The data presented here suggest that the risk for zoonotic transmission of PARV4-like viruses from nonhuman primates in West Africa is low. Nonetheless, parvovirus evolution has pointed toward occasional cross-species transmissions (*9*). This observation, coupled with the frequent intimate contact between bushmeat hunters and their prey, compels the continued vigilance for cross-species transmission of these viruses and others with the intention of mitigating the risk posed by novel introductions of viral zoonoses.
